# Destruction of *Staphylococcus aureus* and the impact of chlortetracycline on biomethane production during anaerobic digestion of chicken manure

**DOI:** 10.1016/j.heliyon.2019.e02749

**Published:** 2019-11-14

**Authors:** M.E. Kirby, M.W. Mirza, T. Leigh, L. Oldershaw, M. Reilly, S. Jeffery

**Affiliations:** a Agricultural Centre for Sustainable Energy Systems (ACSES), Animal Production, Welfare and Veterinary Sciences Department, Harper Adams University, Newport, Shropshire, TF10 8NB, United Kingdom; b Princess Margaret Science Laboratories, Harper Adams University, Newport, Shropshire, TF10 8NB, United Kingdom; c Crop and Environmental Sciences Department, Harper Adams University, Newport, Shropshire, TF10 8NB, United Kingdom

**Keywords:** Microbiology, Bioenergy, Wastewater management, Antibiotics, Anaerobic digestion, *Staphylococcus aureus*, Chlortetracycline, Biochemical methane potential, Chicken manure

## Abstract

Research was undertaken to ascertain the effect on biogas potential during the anaerobic digestion of chicken manure containing *Staphylococcus aureus* and chlortetracycline (antibiotic) from infected chicken flocks. *S. aureus* is a pathogenic bacteria in chicken flocks that is usually treated with the broad-spectrum antibiotic, chlortetracycline. Veterinary antibiotics are often prescribed in the poultry sector for on-farm use at the flock level to control disease; consequently, significant quantities of antibiotics are excreted from the bird into the manure. Subsequent anaerobic digestion of this chicken manure could lead to pathogens and antibiotics affecting the digestion process. Anaerobic digestion biochemical methane potential assays were completed at 35°C for 39 days, with some assays receiving *S. aureus* and some receiving *S. aureus* and chlortetracycline. No viable *S. aureus* cells were detected after Day 0 of the experiment. A further experiment utilising an order of magnitude greater concentration of *S. aureus* demonstrated a significant reduction (>400 fold) in *S. aureus* within 24 h when inoculated into anaerobic digestate, with no viable *S. aureus* cells detected by the end of 3 days. Furthermore, the efficacy of chlortetracycline was significantly reduced when applied to anaerobic digestate compared to water alone. Total biogas yields from chicken manure were significantly lowered by the addition of *S. aureus*, with and without chlortetracycline. However, there was no significant difference in methane yields between treatments. The cellulose control assays showed a lag phase in methane production after receiving chlortetracycline. In comparison, the absence of a lag phase when the antibiotic were added to chicken manure may have been due to the relatively high nitrogen content of the feedstock reducing the inhibition of chlortetracycline on methanogens. Therefore, this study demonstrates that the addition of *S. aureus* and chlortetracycline does not have a commercially relevant effect on the digestion of chicken manure.

## 1 Introduction

Anaerobic digestion (AD) has increased rapidly over recent decades; both in terms of the global number of functioning systems and our technical understanding of the process (Abbasi et al., 2012; Appels et al., 2011; Clarke, 2018). The uptake of AD has been supported, and sometimes driven, by the provision of governmental subsidies. These have incentivised the uptake of AD due to financial returns based on the production of biogas and later conversion to electrical and/or heat energy, including from waste biomass materials (Jones and Salter, 2013).

Different feedstocks lead to differences in the composition and quantity of biogas output when processed through AD (Chynoweth et al., 1993; Gunaseelan, 2004). Biochemical methane potential (BMP) assays are used to determine the maximum quantity of biomethane produced by a feedstock. However, as primarily the carbon in the feedstock is mineralised in the process of methane production, nutrients that were present in the initial feedstock remain in the digestate. Digestate is a term used to describe the residual output that includes the remains of the feedstock along with active and dead microbes that were involved in the AD process. Digestate can then be used for field application to provide plant nutrients and offset the amount of synthetic fertilisers required (Möller and Müller, 2012; Tambone et al., 2010).

An issue with the use of animal wastes or by-products for AD is the potential for pathogen contamination (Mawdsley et al., 1995; Pell, 1997). Research has been undertaken to investigate the survival of pathogens through the AD system and their viability in the resulting digestate (Avery et al., 2014; Manyi-Loh et al., 2014). Results are highly variable and depend on both the pathogen and the AD processing technology (e.g. mesophilic versus thermophilic). Some pathogens, such as *Streptococcus faecalis* can be quickly and effectively destroyed by AD (Olsen and Larsen, 1987). Other pathogens, such as *Clostridium* sp*.,* which are spore formers, are much more resistant and can remain viable in the resulting digestate (Sahlström, 2003; Kirby et al., 2018).

The identification of pathogens, such as *S. aureus,* within a commercial poultry setting can lead to the prescription and administration of antibiotics to control the infection; within the UK usually chlortetracycline. This antibiotic has been shown to survive passage through the intestinal tract of birds and to remain active in the litter, where it can reach concentrations of 66 mg/kg of litter (Furtula et al., 2010). Previous work has suggested that *S. aureus* does not survive the AD process well, with data suggesting complete kill of the bacteria within the first day of the AD process (Olsen and Larsen, 1987). This speed of deactivation is surprising considering *S. aureus* is a gram positive, facultative anaerobe and therefore it could be hypothesised to survive for longer within an AD system. The variation in the lengths of times that which have been reported for deactivation of *S. aureus* within AD systems warrants investigation as it is a common poultry infection and zoonotic. Furthermore, the impacts of antibiotics on the AD process are not well understood. Research has demonstrated that the presence of low concentrations of chlortetracycline (<60 mg/kg-total solids (TS)) can have a positive increase in biogas generation as the chlortetracycline is degraded and the carbon and nitrogen present within the antibiotic is reused for microbial growth (Yin et al., 2016). However, at higher concentrations, chlortetracycline can inhibit methanogenesis (Yin et al., 2018). As such, it remains an open question as to whether AD system engineers should be informed to avoid feedstocks that may contain residual antibiotics, or whether those antibiotics are deactivated by the AD process such that they do not impact on biogas yields.

Here we investigate a realistic scenario for AD within the UK poultry sector by using a time-series kill BMP assays on chicken manure feedstock with and without the addition of *S. aureus* and chlortetracycline. Through the use of microbiological culturing techniques, we aim to test the following hypotheses:

• AD of chicken manure will decrease the viable population of *S. aureus* over time
• The presence of chlortetracycline in the chicken manure fed to anaerobic digesters will expedite the decrease in viable populations of *S. aureus* over time
• The presence of chlortetracycline in the chicken manure will decrease the biogas yield of the AD process

## 2 Materials and methods

### 2.1 Experimental design

The BMP assays were operated at 35°C, with eight sequential kill dates undertaken on Days 0, 1, 2, 4, 8, 16, 32 and 39 (end of experiment). At each time kill, the bottles were unsealed using a de-crimping tool and analysed for TS, volatile solids (VS) and *S. aureus* viable counts. The experimental treatments were Chicken manure only; Chicken manure with the addition of *S. aureus* and Chicken manure with the addition of *S. aureus* and chlortetracycline (at 66.2 mg/kg from Furtula et al., 2010). Additionally, cellulose controls and blank assays were included, Blank – digestate only; Control – digestate and cellulose only; Separate blanks and controls with the addition of *S. aureus*; Separate blanks and controls with the addition of chlortetracycline; Separate blanks and controls with the addition of *S. aureus* and chlortetracycline.

All BMP assays were replicated three times, totalling 96 BMP assays. BMP assays were loaded on a VS ratio of 2:1 (inoculum:chicken manure), with the total working volume standardised across all treatments by the addition of distilled water. All BMP assays were allocated a position in the incubator by using a random number generator in Excel.

### 2.2 Preparation of BMP components

Prior to the start of the experiment, 10 L of sewage sludge digestate were collected from a local sewage works (Severn Trent, Shropshire, UK) and sieved (600 μm) to remove larger particles. The sewage sludge was left at room temperature to degas for seven days. Chicken manure (∼200 g) was collected from a layer chicken shed that had not received antibiotics (Oaklands Farm Eggs, Shropshire, UK) and was refrigerated (4°C) until use.

### 2.3 Experimental routine

Chicken manure and sewage sludge were analysed for TS and VS content prior to the start of the experiment to determine loading volumes for the BMP assays. The BMP assays were loaded as shown in Table 1. Chlortetracycline was added to a final concentration of 66.2 mg/l. BMP assays were inoculated to an initial level of 10^5^ colony-forming units (CFU)/ml of *S. aureus*.

**Table 1:** Biochemical methane potential assay loadings per treatment.

Treatment	Digestate (ml)	Chicken manure (g)	Cellulose addition (g)	*S. aureus* addition (ml)	Chlortetracycline addition (ml)	Water (ml)	Total volume (ml)
Chicken manure	45.00	1.94	-	-	-	1.06	48.00
Chicken manure and *S. aureus*	45.00	1.94	-	0.05	-	1.01	48.00
Chicken manure, *S. aureus* and chlortetracycline	45.00	1.94	-	0.05	1.00	0.01	48.00
Control – cellulose	45.00	0.00	0.51	-	-	2.44	48.00
Control – cellulose and *S. aureus*	45.00	0.00	0.51	0.05	-	2.44	48.00
Control – cellulose and chlortetracycline	45.00	0.00	0.51	-	1.00	1.44	48.00
Control – cellulose, *S. aureus* and chlortetracycline	45.00	0.00	0.51	0.05	1.00	1.44	48.00
Blank – digestate only	45.00	0.00	-	-	-	3.00	48.00
Blank – *S. aureus*	45.00	0.00	-	0.05	-	2.95	48.00
Blank – chlortetracycline	45.00	0.00	-	-	1.00	2.00	48.00
Blank – *S. aureus* and chlortetracycline	45.00	0.00	-	0.05	1.00	1.95	48.00

After each BMP assay was compiled, the bottles were sealed, degassed with nitrogen for 10 min to remove any oxygen present in the headspace and equalised to atmospheric pressure. The bottles were then placed in a pre-warmed incubator (35°C). The biogas pressure and volume were measured as required throughout the experiment. Biogas composition (methane and carbon dioxide) was measured using a micro-gas chromatograph (GC) (see Section 2.4). At each kill time, bottles were agitated manually to ensure a homogenous sample and this was followed by biogas, TS, VS and *S. aureus* sampling.

### 2.4 Chemical analyses

Biogas analyses were undertaken using an Agilent 490 micro-GC, with dual columns (molsieve column for methane and a CP-PoraPLOT U column for carbon dioxide). The injector and columns were heated to 60°C and 80°C respectively, with the biogas sampled for 10 ms onto the molsieve (backflush set to 34 s) and 100 ms for the PoraPLOT. The sample was transferred using helium and analysed for 115 s at a pressure of 120 kPa. The TS and VS content of the feedstocks and digestate samples were using standard methods (APHA, 1989).

### 2.5 Microbial analyses

A stock culture of freeze dried *S. aureus* subsp. *aureus* NCIMB 13062 (NCIMB, Aberdeen, Scotland) was reconstituted following NCIMB guidelines and used to produce source plates on 5% sheep's blood agar. A pure colony of *S. aureus* was inoculated into 10 ml of sterile brain heart infusion broth, in duplicate, and incubated at 37°C for 24 h with agitation at 150 rpm. Following incubation, the culture was serial diluted to 10^−8^, in duplicate, and plated onto mannitol salt agar (MSA). Agar plates were incubated for 72 h at 35°C and the colonies counted. Additionally, a stock solution of chlortetracycline (Sigma-Aldrich, UK) with a concentration of 3,310 mg/l was made and sterilised by passing through a 0.2 μm filter, prior to storing in a sterile plastic container at 4°C before use.

**Fig. 1:**
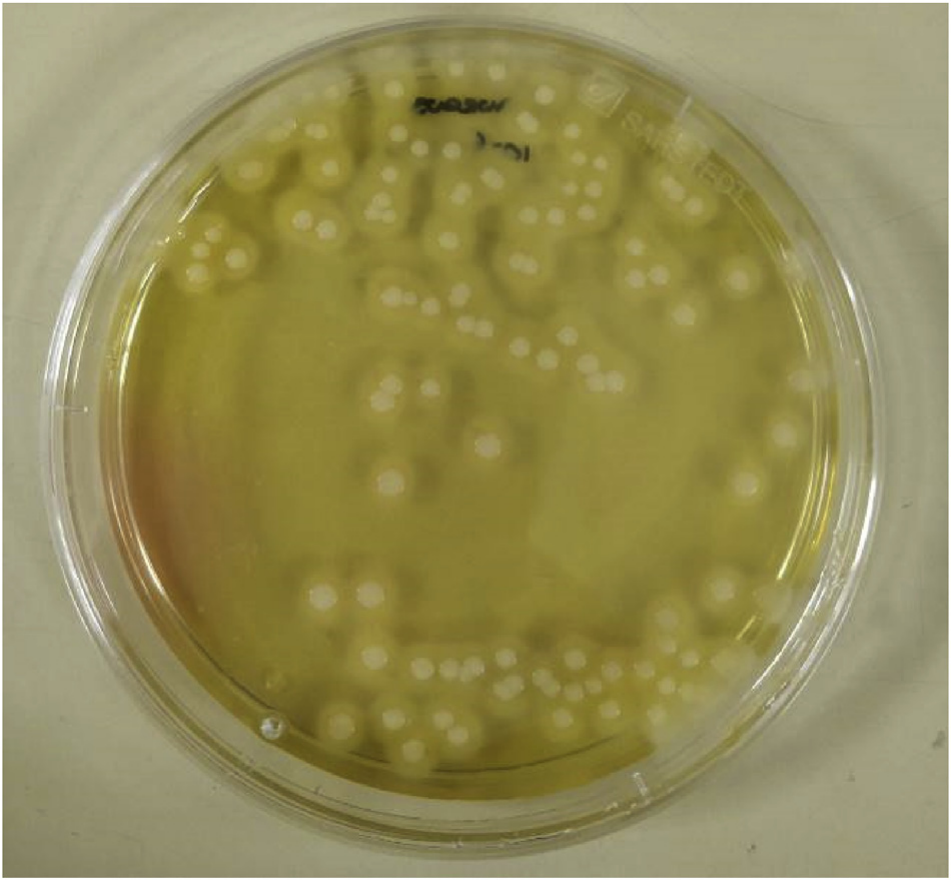
Mannitol salt agar plate inoculated with *Staphylococcus aureus*. Yellow colouration of the agar and haloes are visible surrounding the colonies.

Initial counts of *S. aureus* present in chicken manure and sewage sludge were performed. One gram of chicken manure was aseptically weighed into a sterile container and 9 ml of sterile maximum recovery diluent (MRD) (ThermoFisher Scientific, Waltham, WA) was added. The manure and diluent suspension was agitated by vortex mixer to produce a homogenous suspension. The suspension was serially diluted in MRD to a dilution factor of 10^−8^. From each suspension, 100 μl was pipetted onto sterile MSA and aseptically spread over the agar surface using a sterile plastic spreader. The MSA plates were then inverted and incubated at 35 °C for 72 h. Following incubation, the plates were examined for the presence of a yellow colour indicated by the agar and colonies with surrounding ‘haloes’ (illustrated in Fig. 1). Colonies were counted and viable counts of *S. aureus* calculated. The same determination was performed on the sewage sludge using 1 ml of sample in 9 ml of sterile MRD. For *S. aureus* determination at each kill day, 1 ml sample was extracted and diluted in 9 ml of sterile MRD and cultured as previously described.

#### 2.5.1 Persistence of *S. aureus* confirmation sub-experiment

To maximise the temporal resolution early on in the AD process a sub-experiment was conducted. Nine further bottles were prepared as previously described, three bottles for each kill day. Samples were enumerated for *S. aureus* on Days 0, 1 and 3. Bottles were inoculated to an initial level of 10^6^ CFU/ml of *S. aureus*. Three separate samples were prepared in sterile plastic containers for Day 0 in order to determine if there was a difference in viable counts between glass and plastic sample bottles. *S. aureus* were enumerated following the method described in Section 2.5, using a dilution factor of 10^−8^.

### 2.6 Persistence of antibiotic

A culture of *S. aureus* was prepared as previously described. Chlortetracycline stock solution was added to 10 ml samples of digestate and sterile water into separate centrifuge tubes to a final concentration of 66.2 mg/kg. The samples were manually agitated then centrifuged at 3800 RCF for 15 min. A sample of the supernatant was drawn into a sterile syringe and filter sterilised into a sterile sample tube. 100 μl of the *S. aureus* culture was inoculated onto a sterile Mueller-Hinton agar plate and aseptically spread to produce confluent growth. A sterile paper disk was applied to the centre of the agar plate and 10 μl of the filtered sterilised extract was applied to the disk. Plates were allowed to dry for 10 min before they were inverted and incubated at 35°C for 24 h. Following incubation, the annular radius of the zone of inhibition caused by the antibiotic was measured using digital callipers.

### 2.7 Statistical analyses

Data was analysed by one-way ANOVA to determine the effect of treatment (chicken manure; chicken manure and *S. aureus*; chicken manure, *S. aureus* and chlortetracycline), with three replicates per treatment. Statistical analyses were conducted using GenStat version 18, with a significance level of P<0.05, using Fisher's Least Significant Difference. Analysis of the impact of chlortetracycline was determined by a one-way ANOVA, comparing the radii of the zones of inhibition for chlortetracycline mixed with digestate compared to a water sample.

## 3 Results and discussion

The numbers of viable *S. aureus* in both chicken manure and sewage sludge were found to be below detectable limits. As such, it can be concluded that any *S. aureus* recovered were from the spiked colonies rather than contamination from either the chicken manure or sewage sludge.

No viable *S. aureus* cells were detected after Day 0 in the initial investigation. While this is in agreement with work reported by Olsen and Larsen (1987), in order to further test this result a sub-experiment was run utilising a higher titre of cells with kills on Days 0, 1 and 3. These results, presented in Table 2, confirmed rapid inactivation of *S. aureus* by the AD process with a >400 fold reduction in viable cells within the first 24 h, and no active *S. aureus* cells recovered by Day 3. This result further confirms that the pathogen *S. aureus* is rapidly deactivated by the AD process. Our findings suggest that the probability of any cells remaining active following a hydraulic retention time of >1 week, as per all meso- and thermophilic commercial AD systems, is small to zero.

**Table 2:** Number of viable counts of *Staphylococcus aureus* recovery (colony-forming units (CFU)/ml) from biochemical methane potential assays over the first three days of incubation.

Kill Day	Counted *S. aureus* colonies	Dilution factor	Viable counts (CFU/ml)	Mean viable counts (CFU/ml) per kill day
0	9	1.0 x 10^−4^	9.0 x 10^5^	4.63 x 10^5^
0	23	1.0 x 10^−3^	2.3 x 10^5^	
0	26	1.0 x 10^−3^	2.6 x 10^5^	
1	10	1.0 x 10^−1^	1.0 x 10^3^	1.07 x 10^3^
1	2	1.0 x 10^−1^	2.0 x 10^2^	
1	20	1.0 x 10^−1^	2.0 x 10^3^	
3	0	0.0 x 10^0^	0.0	0
3	0	0.0 x 10^0^	0.0	
3	0	0.0 x 10^0^	0.0	

**Fig. 2:**
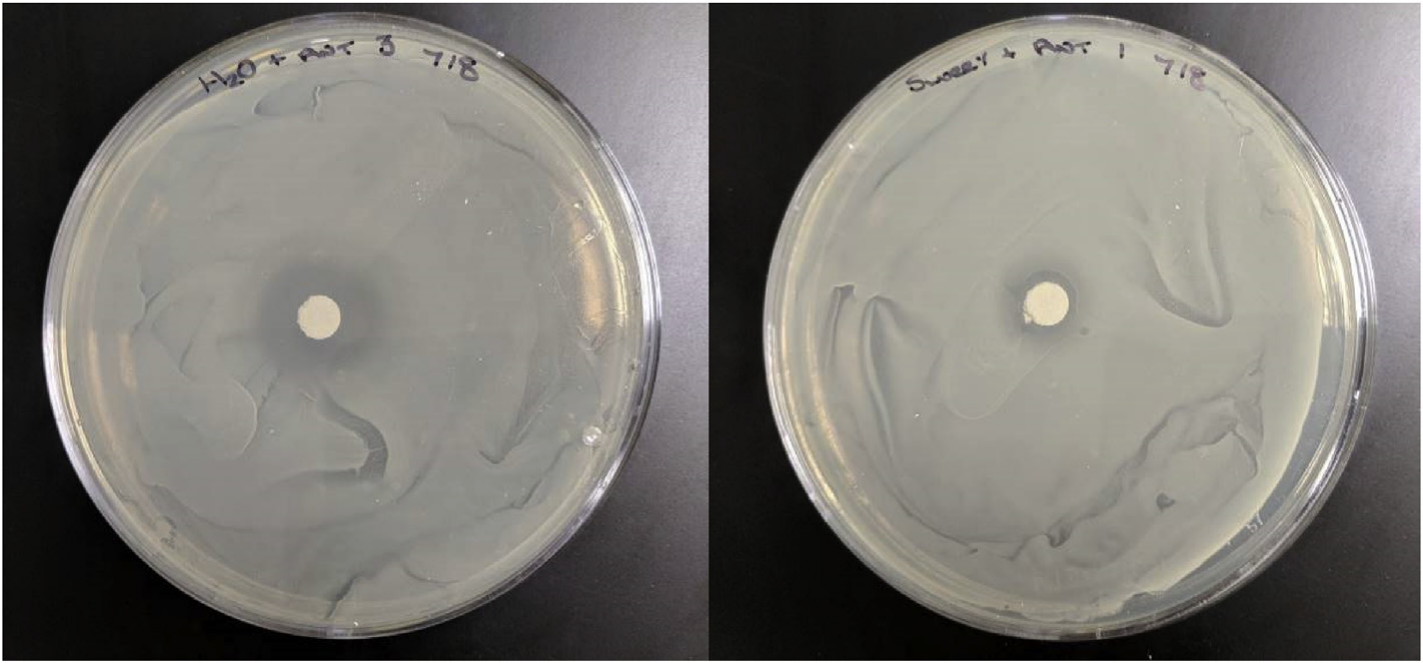
The efficacy of chlortetracycline for inhibiting *Staphylococcus aureus* when chlortetracycline is mixed with either sterile water (left), or anaerobic digestate (right).

The impact of chlortetracycline was significantly (P<0.001) reduced when applied to digestate compared to being mixed with water (Table 3 and Fig. 2). The *S. aureus* zone of inhibition were significantly smaller when mixed with anaerobic digestate, demonstrating the positive effect of AD on reducing the active functioning of chlortetracycline. The mechanism for this reduction remains unclear but there are likely to be three different modes of action that may have reduced the efficacy. Firstly, sorption of the antibiotic onto the organic material present in the digestate can occur, reducing the antibiotic's efficacy (Yin et al., 2016). However, sorption of antibiotics onto organic material is reversible and can lead to future soil contamination (Spielmeyer, 2018). Secondly, the digestate would have contained numerous other microbes. Chlortetracycline is a broad-spectrum antibiotic that could be degraded by other microbes present in the digestate; some bacteria are able to remove the NH_2_ and OH groups of the chlortetracycline, biodegrading the antibiotic (Yin et al., 2016) and using the antibiotic as a source of carbon and nitrogen (Liao et al., 2017). However, Spielmeyer (2018) reported that the degraded compounds from chlortetracycline might still be microbiologically active. Thirdly, it may be that the conditions present in the digestate deactivated the antibiotic by causing structural change. Isomerisation of chlortetracycline is possible under neutral to mild alkaline conditions, similar to the digestate (Yin et al., 2016). As the specific mechanisms by which the efficacy of the chlortetracycline is still not fully understood (Spielmeyer, 2018), this will be key in determining the generality of its efficacy reduction across AD systems. The identification of these mechanisms should be subject to further research.

**Table 3:** Mean annular radius of zones of inhibition of chlortetracycline applied to anaerobic digestate samples and water samples on *Staphylococcus aureus* culture over a three-day period.

Replicate	Annular radius of zone of inhibition (mm)		SEM	P-value
	Water sample	Anaerobic digestate sample		
1	5.7	1.8	0.267	<0.001
2	5.9	2.3		
3	4.8	2.3		
Mean values	5.47^b^	2.13^a^		

SEM - Standard error of the mean.

Total biogas yields were significantly (P=0.005) higher for the chicken manure treatment, with significantly lower yields produced from chicken manure and *S. aureus* and the lowest yield produced from chicken manure, *S. aureus* and chlortetracycline (Table 4). It can be postulated that the addition of *S. aureus* caused a microbial population change reducing total biogas yield. However, the exact mechanism behind this reduction is unknown and requires further research. There was no significant increase in methane yield between the treatments, which for commercial operation suggests that the presence of *S. aureus* with/without chlortetracycline has no significant effect on methane yields produced (Table 4). There was a significantly (P=0.004) higher carbon dioxide yield produced for chicken manure treatments, but this is negligible to commercial AD activities (Table 4). In addition to the chicken manure treatments, the cellulose controls demonstrated that the total biogas yield was significantly (P=0.005) higher for cellulose and cellulose and *S. aureus* assays, with any addition of chlortetracycline reducing the yield. The cellulose and chlortetracycline assay produced significantly the lowest total biogas yield of all control cellulose assays (Table 5). There were no significant effects on methane and carbon dioxide yields (Table 5). Other published works have demonstrated variable effects on biogas yields when digesting chlortetracycline. Yin et al. (2016) demonstrated an increase in biogas yields at lower concentrations of chlortetracycline (<60 mg/kg-TS). However, other authors have noted a variable and significant decrease in biogas yields (>62%) at a range of inclusion levels (Álvarez et al., 2010). Here, using a high but realistic rate we show that chlortetracycline had no significant impact on BMP within the parameters used in this experiment.

**Table 4:** Mean total biogas, methane and carbon dioxide yields (ml/g-VS-fed) for chicken manure digested with and without the addition of *Staphylococcus aureus* and chlortetracycline.

Biogas yields (ml/g-VS-fed)	Treatments			SEM	P-value
	Chicken manure	Chicken manure and *S. aureus*	Chicken manure, *S. aureus* and chlortetracycline		
Total biogas	450.4^c^	434.0^b^	416.9^a^	4.40	0.005
Methane	223.5	220.1	211.0	4.64	0.223
Carbon dioxide	107.5^b^	100.5^a^	96.9^a^	4.60	0.004

SEM - Standard error of the mean. Mean data in rows with the same superscript are not significantly different (P>0.050).

**Table 5:** Mean total biogas, methane and carbon dioxide yields (ml/g-VS-fed) for cellulose controls with and without the addition of *Staphylococcus aureus* and/or chlortetracycline.

Biogas yields (ml/g-VS-fed)	Treatments				SEM	P-value
	Cellulose	Cellulose and *S. aureus*	Cellulose and chlortetracycline	Cellulose, *S. aureus* and chlortetracycline		
Total biogas	751.9^c^	740.9^bc^	713.4^a^	724.6^ab^	5.32	0.004
Methane	381.4	382.0	350.2	345.9	9.71	0.051
Carbon dioxide	268.4	275.1	278.0	285.4	6.31	0.355

SEM - Standard error of the mean. Mean data in rows with the same superscript are not significantly different (P>0.050).

**Fig. 3:**
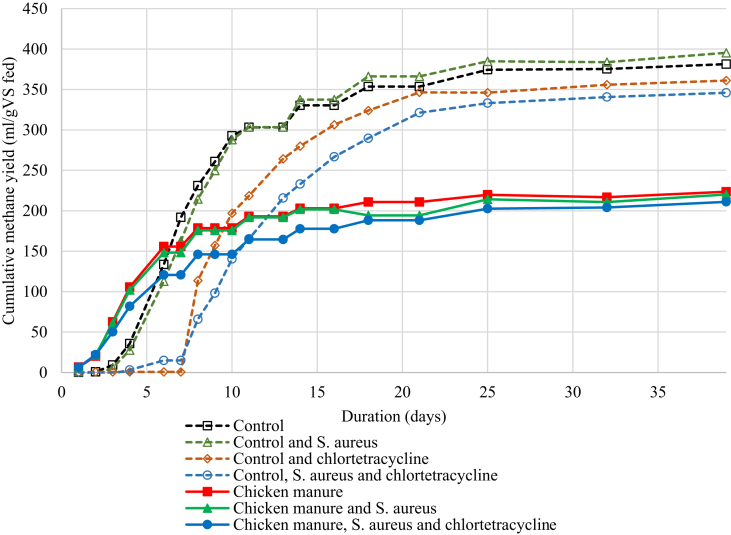
The mean cumulative methane yield (ml/g-VS-fed) from cellulose and chicken manure following subtraction of methane production from digestate (blank) controls.

It can be seen in the cumulative biogas production (Fig. 3) that the rate of methane production varied over time and between treatments. All treatments containing chicken manure had a similar pattern of methane production across time, with no inhibition or lag phase noted with the addition of *S. aureus* and chlortetracycline. The methane yields (ml/g-VS-fed) for chicken manure treatments were similar to other published data (Kafle and Chen, 2016). The cellulose controls produced the expected methane yields, but the controls that contained chlortetracycline exhibited a lag phase in methane production until Day 7. This lag phase demonstrates an inhibitory effect of the chlortetracycline on microbial populations, before the antibiotic was degraded or absorbed by surrounding organic matter. These lag phases were not observed in the chicken manure treatments receiving chlortetracycline. This may have been due to the increased nitrogen content from the treatments receiving chicken manure, increasing the biodegradation rate of the chlortetracycline as previously described by Liao et al. (2017).

## 4 Conclusion

This research demonstrates that AD is an effective method at destroying *S. aureus* populations. The efficacy of chlortetracycline is reduced when mixed with anaerobic digestate. Total biogas yield was significantly reduced when chicken manure was digested with *S. aureus* and chlortetracycline; however, there was no significant effect on methane yields. The rate of methane production was consistent across all chicken manure treatments; there was a distinctive lag phase (7 days) in methane production from the cellulose controls that contained chlortetracycline. This absence of a lag phase for chicken manure treatments may be attributed to the higher nitrogen content of the chicken manure increasing the biodegradation rate of chlortetracycline.

## Declarations

### Author contribution statement

Kirby, ME: Conceived and designed the experiments; Performed the experiments; Analyzed and interpreted the data; Contributed reagents, materials, analysis tools or data; Wrote the paper.

Mirza, MW, Oldershaw, L, Reilly, M: Conceived and designed the experiments; Performed the experiments; Contributed reagents, materials, analysis tools or data.

Leigh, T: Conceived and designed the experiments; Performed the experiments; Analyzed and interpreted the data; Contributed reagents, materials, analysis tools or data.

Jeffery, S: Conceived and designed the experiments; Analyzed and interpreted the data; Wrote the paper.

### Funding statement

This work was supported by the European Union's Horizon 2020 research and innovation programme as part of the project AgroCycle (Grant agreement No 690142).

### Competing interest statement

The authors declare no conflict of interest.

### Additional information

No additional information is available for this paper.
